# Augmenting Traditional Cardiac and Medical Care in Africa via Telemedicine: A Pilot Study

**DOI:** 10.7759/cureus.17483

**Published:** 2021-08-27

**Authors:** Okechukwu N Mgbemena, Isaac Sears, Barry Levine

**Affiliations:** 1 Cardiology, University of Florida, Jacksonville, USA; 2 Medicine, University of Virginia, Charlottesville, USA; 3 Computer Science Department, San Francisco State University, San Francisco, USA

**Keywords:** teleconsultation, sub-saharan africa, telemedicine (tm), nigeria, cardiac care

## Abstract

Background

A critical decrease in the number of healthcare providers in developing countries is one of the major burdens to healthcare access in these countries. Many factors contribute to the lack of healthcare providers, including low doctor-to-population ratio, emigration of doctors to other countries, long travel distances to hospitals, increasing cost of healthcare, and concentration of doctors in urban cities. Several measures have been taken by both governmental and nongovernmental organizations in these countries to mitigate this crisis with varying outcomes. In this study, we investigate the use of technology in the form of telemedicine in a developing country. We evaluate patient predisposition to the use of telemedicine, their experience, and some challenges involved in the use of telemedicine in this setting.

Methodology

We set up an electronic medical record system, OpenMRS, and added telemedicine modules to the system. Then, we recruited doctors and gave them privileges on OpenMRS after carefully vetting their credentials. Finally, we set up a website through which patients could request telemedicine consultations. We registered a telephone number in Nigeria so that patients could also request consultations via SMS. Consult requests were then entered into OpenMRS. Doctors logged in periodically and checked for patients awaiting consults. They called patients, diagnosed them, requested further diagnostics, and/or sent prescriptions to patients via SMS directly from OpenMRS.

Results

Data were collected over the first year of telemedicine service in Nigeria. These data were then analyzed to understand the effectiveness, patient experience, cost efficiency, and general utilization of this service. In the first year, there were 510 new patient registrations and 572 total consultations. Patient age ranged from less than one year to 77 years, with a median of 29 years. Among the users of the service, 51.8% (264) were female. For consult requests, 52.2% of requests were via the web, and others were via SMS requests. There were over 50 reviews of the service on the website and social media, and 95% of users reported a positive experience.

Conclusions

From preliminary data, telemedicine can potentially be a good adjunct to help doctors reach their patients, especially in rural areas where there is an immense shortage of healthcare professions. Although most patients reported a positive experience, further investigations are needed to validate our experience.

## Introduction

A critical decrease in the number of healthcare providers in developing countries is one of the major burdens to healthcare access in these countries. This deficit is most severe in Africa which holds 11% of the world’s population [1]. Many factors contribute to the lack of healthcare providers, including low doctor-to-population ratio [2], emigration of doctors to other countries [2], long travel distances to hospitals [3], increasing cost of healthcare, and concentration of doctors in urban cities [1]. Africa has only 2.3 healthcare workers per 1,000 population in contrast to most developed countries, for example, the United States which has 24.8 healthcare workers per 1,000 population [2]. The shortage of healthcare workers in developing countries undoubtedly contributes to poor healthcare outcomes and widespread disease burden resulting in decreased life expectancy and quality of life [4]. Several measures have been taken by both governmental and nongovernmental organizations in these countries to mitigate this crisis with varying outcomes. Like the problem itself, the solution is multifaceted and involves both political and economic commitment to healthcare in these countries. In settings where the distance between healthcare providers and patients impedes quality or access to care, it has been suggested that telemedicine can potentially be a useful tool in bridging this gap [5]. Several challenges are involved in the setup and operation of the telemedicine service in developing countries. Some of these challenges include lack and cost of infrastructure, lack of collaboration between physicians and information technology personnel, and legal and policy barriers [6]. Interestingly, the availability and use of mobile phones and access to the internet have been on the rise in developing countries, making telemedicine a more attractive adjunct for these patients. In this study, we aim to investigate the use of technology in the form of telemedicine in a developing country as an adjunct to traditional cardiac and medical practice. We evaluate patient predisposition to the use of telemedicine and their experience.

## Materials and methods

We set up an electronic medical record system, OpenMRS, on a secure and encrypted cloud server and added telemedicine modules to the system mainly consisting of a consult list where patients awaiting consultations were posted. Then, we recruited 12 doctors through public advertisement and gave them privileges on OpenMRS after careful vetting of credentials. Doctors and ancillary staff were trained via video demonstrations on how to use the system. They were trained on how to write consult notes, send electronic diagnostic requests (e.g., labs, X-rays, etc.) to patients, and how to send electronic prescriptions to patients directly from OpenMRS. Finally, we set up a website, https://www.fortitudotelemed.com/, through which patients could request telemedicine consultations. For patients who may not be tech-savvy or may prefer to request a consultation through other means, we registered a telephone number in Nigeria so that they could request consultations via SMS. After requests were received, we enter patient’s information on a consult list on OpenMRS. Finally, if necessary, patients could submit pictures (e.g., dermatologic concerns) or other documents including laboratory and radiologic results. This additional information was also uploaded to the patient’s record where doctors could view them. The telemedicine service received by patients was completely free of charge to them and was available to patients from several African countries where legislation is not prohibitive.

**Figure 1:**
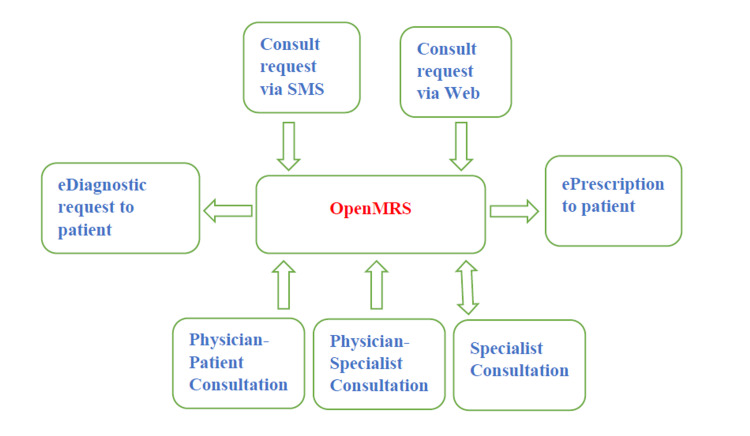
Schematic of the operational flow of Fortitudo Telemedicine.

Doctors logged in periodically or on receiving notifications on a secure group chat to check for patients awaiting consults. They called patients (on a first-come-first-served basis), diagnosed them, requested further diagnostics, and/or sent prescriptions to patients via secure SMS from OpenMRS. Patients completed their diagnostic tests at any facility of their choice (with no affiliation to this telemedicine service), and when results were available, they were sent to their requesting doctor for review. When treating doctors needed a specialist perspective on a patient, they could request a physician-to-physician consultation with a specialist associated with the project Connecting Kids with Care (CKC) [7]. CKC is a free resource that connects organizations working with vulnerable populations to volunteer licensed medical professionals for a physician-to-physician consultation. Our OpenMRS system interoperates with CKC to facilitate prompt physician-to-physician consultations on patients who need a specialist (Figure 1).

After the consultation was completed and the patient received ePrescription and/or diagnostic requests, they could take the SMS requests/prescriptions to their pharmacy and/or laboratory/diagnostic center to obtain medications or for further testing, respectively.

## Results

We collected data on the utilization of telemedicine service in the first year from May 2018 to May 2019. Over this time period, there were 510 new patient registrations and 572 telemedicine consultations. Among patients, age ranged from less than one year to 77 years with a median age of 29 years (mean age: 30.8 years). Of the 510 newly registered patients, 51.8% (264) were female (Table 1). The rate of repeat consultation was 10.8% and did not differ between male and female patients. The peak age group of users was 21-30 years, which constituted 46.5% (237) of all users. Among users, 52.2% (266) requested a consultation via our website, while 47.8% (244) requested a consultation via mobile SMS. There was no statistically significant difference with respect to sex among patients who used the website to request a consult (52.3% (139) male vs. 47.7% (127) female). Most website users (72.8%) preferred to get service from any available doctor, while 27.2% indicated that they preferred a specific doctor. To get a measure of physician responsiveness, we measured time-to-complete charting. The median time-to-chart for all patients was 102 minutes. Among all registered patients, only 1.1% (six) were unreachable by telephone.

**Table 1:** Demographics and utilization of telemedicine service (weighted sample, N = 510).

Ages	%
Age: 0-10	3.9%
Age: 11-20	6.7%
Age: 21-30	46.5%
Age: 31-40	27.8%
Age: 41-50	8.8%
Age: 51-60	3.3%
Age: 61-70	2.2%
Age: 71-80	0.59%
Sex	
Female	51.8%
Male	48.2%
Medium of consult request	
Via website	52.2%
Via SMS	47.8%
Prescription and diagnostic requests	
Electronic prescriptions	81.2%
Request for further diagnostics	54.7%

We also analyzed the cost involved in running a telemedicine service. During the first year of service, the total cost of providing 572 consultations was $3,537.09, bringing the overall cost per consult to $6.18. This overall cost included the cost of the initial infrastructure setup, company registration, and other miscellaneous expenses. Month-to-month “running cost” (excluding initial setup cost and after expenditure stabilization) averaged to $3.31/consult. As this pilot study was funded by donations, the service was provided free of charge to patients. Expenditure was further broken down by type: physician/clerk cost which included the amount paid to doctors and clerks for providing services; and advertisement cost which included the cost of advertisement for the service on social media, SMS campaigns, and printing/distribution of flyers. Finally, technology included the cost of OpenMRS host server, website, and domain purchase/maintenance.

The majority of the cost was for advertisement (41.5%), followed by technology (36%) and physician/clerk (22.5%). We also analyzed the proportion of consults by doctor type. Most consults (85.1%) were done by paid physicians, while nonpaid, volunteer physicians completed 14.9% of consultations. Table 2 shows the breakdown of expenditure by type and doctors who completed consults by type.

**Table 2:** Breakdown of expenditure by type and doctors who completed consults by type of expenditure and doctors.

Expenses	%
Physician/clerk cost	22.5%
Advertisement cost	41.5%
Technology	36%
Consult by doctor type	
Paid doctors in Nigeria	85.1%
Nonpaid volunteer doctors abroad	14.9%

## Discussion

Telemedicine service is an emerging technology in most developing countries and can potentially be a great adjunctive tool to bridge the gap in healthcare access in developing countries [8]. Currently, Africa is one of the most underserved continents from a healthcare infrastructure standpoint due to low physician density, lack of advanced medical technology, and lack of trained personnel [9]. Due to pervasive poverty, patients in these countries do not have access to quality healthcare, and when there is quality healthcare, most of the population is unable to afford it. Emigration of healthcare workers from Africa and the resultant low physician-to-population ratio is also a major contributing factor to the deficits in this region [10]. The physician-to-population ratio in most middle-income and low-income countries in Africa is below the 1/5,000 World Health Organization standard [11]. This low physician-to-population ratio is partly due to brain drain, whereby physicians and other healthcare workers in Africa migrate to developed countries to seek better employment opportunities [12]. High-income countries, such as Australia, Canada, the US, and the UK, have sustained a relatively high physician-to-population ratio by recruiting medical graduates from developing regions, including countries in sub-Saharan Africa [13]. Due to these problems, it seems that a reasonable solution to inadequate access to healthcare in Africa must include a readily available, affordable, and accessible means of healthcare to patients living in these countries. There have been many governmental and nongovernmental policies that are geared toward addressing the problem of healthcare shortage in Africa. These policies have been met with varying degrees of effectiveness. Technology in the form of telemedicine can potentially transform how healthcare is delivered in Africa, giving more people in remote areas of Africa a reliable source of affordable, accessible, and quality healthcare. Due to the increasing availability of telecommunication mobile phones/devices and the internet, access to healthcare can potentially be a click away for these patients who otherwise would have had difficulty obtaining healthcare.

From our experience, it seems patients are open to receiving care via telemedicine. Though we are yet to compare the efficacy of telemedicine to traditional service, we suspect telemedicine may provide more access due to the relatively fast service and the associated convenience to patients. In the first year of service, Fortitudo Telemedicine provided 572 consults to 510 newly registered patients. Most users were in the 21-40-year age group and there was no sex predilection among users. Patients requested consults via our website, https://www.fortitudotelemed.com/, or via SMS to a local phone number. There was no preference among users for web requests versus SMS requests. Among patients who provided feedback, 95% reported having a positive experience with the service.

There are several challenges involved in the setup and operation of the service. As the telemedicine service was provided at no cost to the patients, there was a significant skepticism among patients about the authenticity of the service. This skepticism initially discouraged patients from using the service but later improved as patients started receiving service and were reassured of its authenticity. Other challenges included the cost of setting up this service. A significant portion of the cost involved technology expenses, including setting up and maintaining a secure server for OpenMRS, the cost involved in social media advertisement for the service, the cost of website and domain management. There was also an immense amount of time and skill required to program the telemedicine modules in OpenMRS. From a physician’s perspective, there was a great deal of positive feedback as the involvement of physicians in the service was flexible and compatible with their work schedule. They were also able to follow up with their clinic patients via telemedicine, especially patients who would have had difficulty attending a follow-up visit in the clinic due to distance or other extraneous factors. Another challenge involved clinical presentations that could not be adequately served through telemedicine, as is the case for most medical emergencies. These patients were referred to the nearest hospital for in-person evaluation and treatment. Finally, among doctors who provided the service, paid doctors provided most of the consultations compared to unpaid volunteer diaspora doctors, raising concerns for the long-term financial sustainability of the project as patients currently receive the service free of charge.

Our study has several limitations. The study participants obtained teleconsultations without any charge. It is unclear if patients would be willing to pay for the service if it was not free. The study also had a relatively short duration and a limited patient population in one country, so long-term exploration may be necessary.

## Conclusions

Telemedicine can be a helpful tool to improve healthcare in developing countries as it has the potential to improve healthcare access, provide rapid service, and decrease cost and travel compared to traditional service. Preliminary data suggest that patients are open to this model; however, further data will be needed to validate our experience.
